# Effects of fasting hyperglycemia in men on pregnancy outcomes of singleton pregnant women with cryo-thawed embryo transfer

**DOI:** 10.1186/s40001-023-01591-9

**Published:** 2023-12-19

**Authors:** Li Yang, Xiangming Tian, Huanhuan Li, Junjian Sun, Wenhui Zhou

**Affiliations:** 1 Medical Center for Human Reproduction, Beijing Chao-Yang Hospital, Capital Medical University, Beijing, People’s Republic of China; 2 Department of Obstetrics and Gynecology, Beijing Tongzhou Maternal and Child Health Care Hospital, Beijing, People’s Republic of China; 3 Beijing First Hospital of Traditional Chinese Combined With Western Medicine, Beijing, People’s Republic of China

**Keywords:** Cryo-thawed embryo transfer, Fasting hyperglycemia, Clinical pregnancy outcomes, Live birth, ART outcomes

## Abstract

**Background:**

The relationship of metabolic issues to pregnancy outcomes during assisted reproductive technology (ART) is gaining much attention. Fasting Plasma Glucose (FPG) is one of the most common metabolic indicators. Abnormal FPG not only affects the quality of life of human body, but also has a bearing on reproductive health. However, most attentions are paid on women's physical health and reproductive assessment, the health status of the male partner on pregnancy outcomes during ART treatment is often neglected. This study investigated whether male fasting hyperglycemia (FH, FPG > 6.1 mmol/L) can affect live birth rates (LBR) in singleton intrauterine clinical pregnancy women with cryo-thawed embryo transfer (CET) cycles.

**Material and methods:**

A retrospective cohort study (370 CET cycles with first singleton clinical intrauterine pregnancy and grouped by male FPG) was conducted to analyze the relationship between male FH and clinical pregnancy outcomes using binary logistic regression; the odds ratios (ORs) and 95% confidence intervals (CIs) were calculated as a measure of relevancy. Live birth rate was the main outcome measure.

**Results:**

The live birth rate (LBR) was significantly lower [58.6% vs. 81.8%, *P* = 0.007, adjusted OR 0.635, 95% CI 0.456–0.884] and miscarriage rate (MR) was significantly higher [41.4% vs. 18.2%, *P* = 0.007, adjusted OR 1.575, 95% CI 1.131–2.195] in the FH group when compared with the Con group. There was no difference in healthy baby rate [88.2% vs. 89.6%, *P* = 0.058, adjusted OR 2.143, 95% CI 0.974–4.716] or abnormal birth weight rate (23.5% vs. 11.8%, *P* = 0.238, adjusted OR 2.859, 95% CI 0.777–10.460] between the FH and control group. No birth defects were observed in the present study.

**Conclusion:**

Male FH is an independent risk factor for lower LBR and higher MR in singleton intrauterine pregnancy women with CET cycles.

## Introduction

Assisted reproductive technology (ART) has indisputably become the main treatment for infertility and earned worldwide attention to the safety of this technique. Due to the unique nature of the pregnancy process, great attentions have been paid to the impact of maternal condition on fetuses and offspring. However, the impact of a male partner, who provides half of the genetic information for the offspring, on the outcomes of pregnancy, especially during ART treatment, is often overlooked [1, 2].

Metabolic syndrome (MS) is a complex group of metabolic disorder syndromes characterized by central obesity, dyslipidemia, hypertension, and hyperglycemia. The worldwide prevalence of MS is not only a pathological basis for cardiovascular disease and diabetes mellitus (DM) but also a potential factor affecting human reproduction [3–6]. Fasting hyperglycemia (FH) is an important risk factor for MS. The widespread incidence of FH is obviously increasing due to changes in lifestyle and diet in recent years [7, 8]. The role of FH on male reproduction is further confirmed in men with diabetes, where fluctuations in insulin and glucose concentrations alter spermatogenesis and male reproductive function on multiple levels, including impaired penile erection, ejaculation, metabolic disturbances in testicular cells, a reduction in total sperm count, impaired sperm motility and sperm morphology [9–11]. A clinical study also showed that hyperinsulinemia in fathers presented poorer embryo quality and lower singleton and twin pregnancy rates [4]. Although the effect of FH on male reproductive function is gradually being noticed [11–13], the relationship between paternal FH and the pregnancy outcome of ART is not well described according to the current studies.

Therefore, in the present study, we performed a retrospective clinical investigation to explore the potential impact of male FH on obstetric and neonatal outcomes in patients with cryo-thawed embryo transfer (CET), which will contribute to a better understanding of the impact of male physical status on pregnancy outcomes with ART treatment and a more comprehensive assessment of the effectiveness and safety of ART treatment.

## Materials and methods

### Study design and patients

A retrospective study was conducted at the Medical Center for Human Reproduction, Beijing Chao-Yang Hospital, Beijing, China. The study was approved by the Human Research Ethics Committee of Beijing Chao-Yang Hospital. Based on the inclusion and exclusion criteria, data from 370 male participants whose spouses obtained their first singleton clinical pregnancy after CET were collected in our center between January 2019 and July 2021.

Briefly, the inclusion criteria were as follows: (1) Female with age between 20 and 42 years, (2) Ultrasound confirmed singleton clinical intrauterine pregnancy with CET cycles, (3) Fasting Plasma Glucose (FPG) and liver and kidney function of the male partner was measured before controlled ovarian hyperstimulation (COH), including aspartate aminotransferase (AST), alanine aminotransferase (ALT), total cholesterol (TC), triglycerides (TG), high-density lipoprotein cholesterol (HDL), low-density lipoprotein cholesterol (LDL), creatinine (Cr) and urea nitrogen (UA). The exclusion criteria were: (1) Body mass index (BMI) (kg/m^2^) < 18.5 or  ≥ 25 for women, (2) Women having an underlying disease with potentially adverse effects on pregnancy outcomes (hypertension, diabetes, other metabolic diseases, hepatitis, abnormal intrauterine structure, diseases associated with abnormal immune status and coagulation abnormalities), (3) Male partner with hepatitis; (4) Cycles with testicular sperm extraction (TESE), testicular sperm aspiration (TESA) or late intracytoplasmic sperm injection (ICSI), (5) Donor cycles.

The Chinese Medical Association Diabetes Branch defines the normal FPG ≥ 3.9 mmol/L and ≤ 6.1 mmol/L, while FPG > 6.1 mmol/L is considered as FH. Thus, all 370 cycles included in this analysis were divided into two groups according to the male partner's FPG values, the FH (*n* = 29) and the euglycemia as Con (*n* = 341) [14, 15].

### Participant preparation

In brief, couples with clinical indications for ART should be examined and evaluated accordingly before treatment. The female partner received routine COH protocols depending on their serum hormone levels, age and ovarian function. After oocyte retrieval, in vitro fertilization (IVF) or ICSI was performed and the embryos cultured in vitro were cryopreserved according to the laboratory routine. CET was performed after the preparation of the endometrium with the employment of a natural cycle, an artificial cycle or a stimulation cycle. To avoid bias, embryo transfer (ET) was conducted by the experienced clinicians under ultrasound guidance. Our previous studies have described in detail the process of IVF/ICSI and endometrium preparation protocols [16, 17].

Male partners should also undergo some physical examination and laboratory evaluation. Physical examinations included specialized anthropometric variables such as male height (meters) and weight (kilograms). Laboratory indications included plasma FPG, AST, ALT, TC, TG, HDL, LDL, Cr and UA levels. All examinations should be done before the woman’s entry into the COH cycles.

### Outcome measures

The primary outcome measured in the present study was the live birth rate (LBR). The secondary outcomes were healthy baby rate (HBR), miscarriage rate (MR), early miscarriage rate (EMR), late miscarriage rate (LMR), preterm delivery rate (PTDR), full-term birth rate (FTBR), and other maternal and neonatal outcomes.

Live birth (LB) was defined as the live newborns born after the 28th week of gestation. Miscarriage was defined as clinical pregnancy loss before the 28th gestational week. Early miscarriage (EM) was defined as clinical pregnancy loss before the 13th gestational week. Late miscarriage (LM) was defined as clinical pregnancy loss between the 13th and 28th gestational week. Preterm delivery (PD) was defined as delivery before the 37th week of gestation. Full-term birth (FTB) was defined as delivery after 37 weeks of gestation. Healthy babies (HB) were defined as delivery after 37 weeks of gestation with a neonatal weight > 2.5 kg and < 4.0 kg without malformations. Very low birth weight, low birth weight, and high birth weight were defined as birth weight < 1.5 kg, < 2.5 kg, and ≥ 4.0 kg, respectively [18–20]. Maternal outcomes were cesarean section status and pregnancy-related complications, including gestational diabetes mellitus, gestational hypertension, placenta praevia, premature rupture of membranes, and postpartum hemorrhage. All delivery and neonatal information were obtained from the electronic medical record.

### Statistics analysis

Data were analyzed via SPSS (v26). Normally distributed continuous data were reported as mean ± standard deviation (SD). Non-normally distributed continuous and categorical data were reported as median (interquartile range) (IQR) or % (number/total), with Kruskal–Wallis nonparametric method or χ^2^ analysis to assess between-group differences, respectively.

Multivariate binary logistic regression analysis was used to quantify the effect of male FH on pregnancy outcomes, including HBR, LBR, MR, EMR, LMR, PTDR, FTBR, pregnancy-related complications, gestation weeks at birth, birth weight and sex ratio. Analysis was adjusted for potential confounders, including age, BMI, ALT, HDL and TG level of men, and age and BMI of women. The ORs and 95% CIs were calculated as a measure of relevancy.

## Results

### Baseline characteristics of patients

Between January 2019 and July 2021, a total of 3688 CET cycles were performed in our Center. There were 3016 cycles excluded due to inconsistent with the inclusion criteria, including the women with not first clinical pregnancy (*n* = 138), implantation failure (*n* = 1681), biochemical pregnancy (*n* = 139), ectopic pregnancy (*n* = 15), an ultrasound indicated twin pregnancy (*n* = 376), data with missing information or incomplete results (*n* = 667).

A total of 672 cycles were available for the first singleton clinical intrauterine pregnancy. Of these, women with abnormal BMI (*n* = 232) or underlying disease (*n* = 9), male partners with hepatitis (*n* = 4) and cycles with TESE (*n* = 2), TESA (*n* = 1) or Late-ICSI (*n* = 2) and donor cycles (*n* = 52) were excluded.

**Fig. 1:**
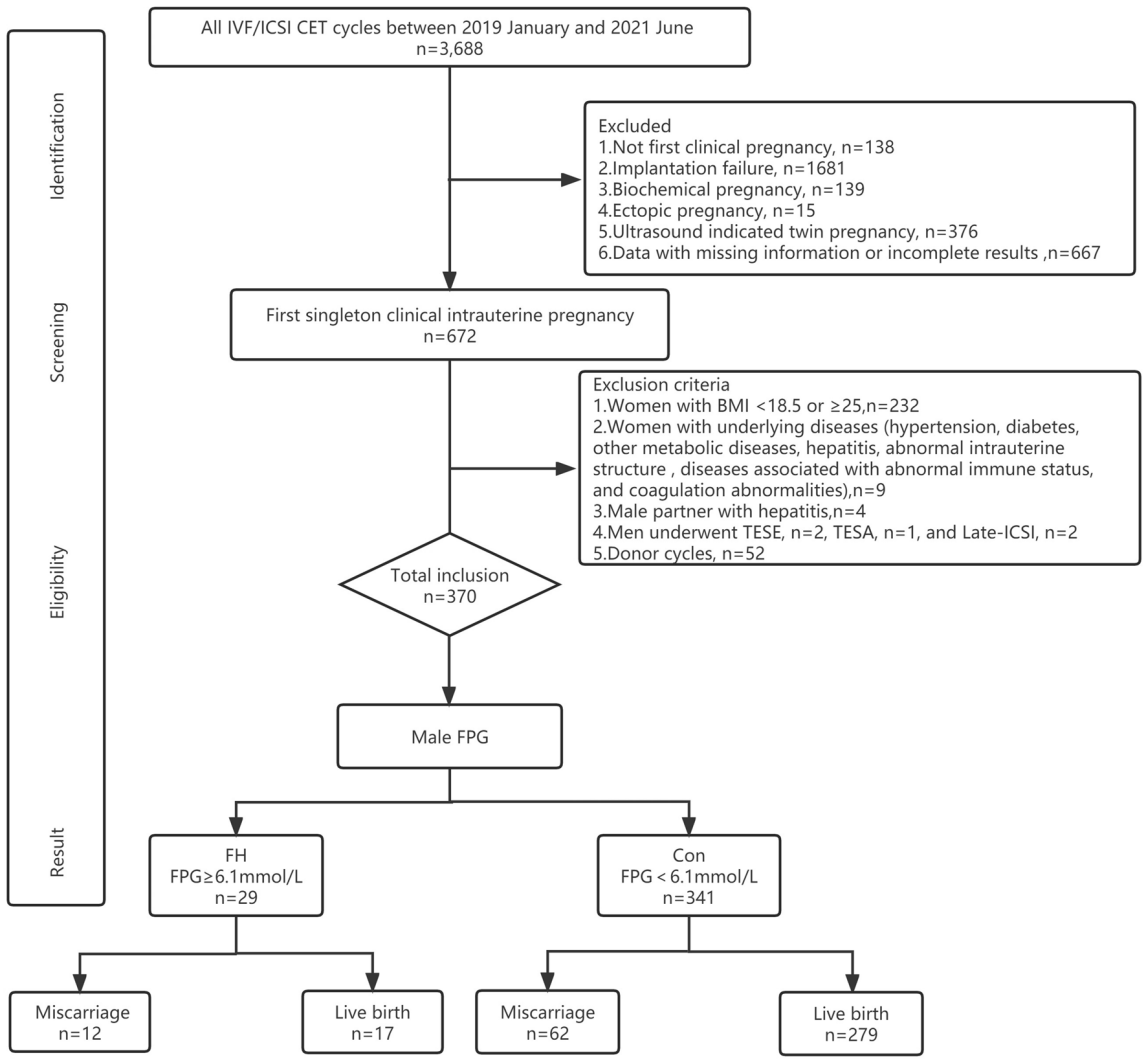
Flowchart of the screening process for the study population. *IVF* in vitro fertilization, *ICSI* intracytoplasmic sperm injection, *CET* cryo-thawed embryo transfer, *FPG* fasting plasma glucose, *FH* fasting hyperglycemia, *TESE* testicular sperm extraction, *TESA* testicular sperm aspiration

The selected 370 eligible cycles were divided into an FH group (*n* = 29) and a normoglycemic control group (*n* = 341) based on the male FPG levels. The obstetric and neonatal outcomes of both cohorts were evaluated to assess the role of male metabolic factors on ART outcomes (Fig. 1).

The baseline clinical characteristic of the participants is shown in Table 1. The age (years) [37.2 ± 5.8 vs. 33.0 (31.0–36.5), *P* = 0.003] and BMI (kg/m^2^) [29.0 ± 3.9 vs. 24.9 (23.2–27.4), *P* < 0.001] of men, woman’s age (years) at CET [32.8 ± 4.3 vs. 32.0 (30–34), *P* = 0.004] and the number of embryos transferred [1(1–2) vs. 2 (1–2), *P* = 0.039] were higher in the FH group compared to the Con group. The proportion of primary infertility (37.9% vs. 62.5%, *P* = 0.009) was lower in the FH group compared to the Con group. No statistical differences were observed in the following parameters, including the woman’s BMI, the number of previous COH and ET cycles, other causes of infertility, years of infertility, number of treatments, fertilization method, development stage of CET embryos, endometrial thickness, morphology and endometrium preparation protocol, between the two groups.

**Table 1:** Baseline characteristics of patients according to FPG

Characteristic	FH (*n* = 29)	Con (*n* = 341)	*P-*value
Male age (y)	37.2 ± 5.8	33.0 (31.0–36.5)	0.003*
Male BMI (Kg/m^2^)	29.0 ± 3.9	24.9 (23.2–27.4)	< 0.001*
< 25 (%)	4 (13.8)	172 (50.4)	
≥ 25 (%)	25 (86.2)	169 (49.6)	
Female age at CET (y)	32.8 ± 4.3	32.0 (30–34)	0.004*
Female BMI (Kg/m^2^)	21.5 ± 1.5	21.5 (20.0–23.1)	0.961
No. of prior COH cycles	1.0 (1.0–1.0)	1.0 (1.0–1.0)	0.130
No. of prior ET cycles	0.0 (0.0–1.0)	0.0 (0.0–1.0)	0.643
Causes of infertility (%)			0.073
Female	20 (69.0)	215 (63.0)	
Male	1 (3.4)	18 (5.3)	
Female and male	2 (6.9)	30 (8.8)	
Others	6 (20.7)	78 (22.9)	
Category of sterility (%) Primary	11 (37.9)	213 (62.5)	0.009*
Secondary	18 (62.1)	128 (37.5)	
Duration of infertility (y)			0.239
< 2 (%)	13 (44.8)	101 (29.6)	
2–5 (%)	13 (44.8)	186 (54.5)	
> 5 (%)	3 (10.3)	54 (15.8)	
Years of treatment (y)	0.7 (0.5–1.2)	0.5 (0.4–0.9)	0.017*
Method of fertilization (%)			0.221
IVF	25 (86.2)	264 (77.4)	
ICSI	4 (13.8)	62 (18.2)	
IVF + ICSI	0 (0)	15 (4.4)	
No. of total embryos transferred	1 (1–2)	2 (1–2)	0.039*
Development stage of CET embryos (%)			0.496
Day 3	14 (48.3)	187 (54.8)	
Day 5–6	15 (51.7)	154 (45.2)	
Endometrium thickness (mm)	9.0 (8.5–10.5)	9.9 (8.1–11.0)	0.700
Endometrium morphology (%)			0.908
A	17 (58.6)	187(54.8)	
A-	8 (27.6)	106 (31.1)	
Others	4 (13.8)	48 (14.1)	
Endometrium preparation (%)			0.434
Natural cycle	11 (37.9)	128 (37.5)	
HRT cycle	17 (58.6)	210 (61.6)	
Stimulation cycle	1 (3.4)	3 (0.9)	

Data are shown as mean ± SD, median (IQR) or *n* (%). The characteristics are the basic patient information routinely provided in ART. *P < 0.05 compared with the control group. *FPG* fasting plasma glucose, *FH* fasting hyperglycemia, *BMI* body mass index, *COH* controlled ovarian hyperstimulation, *ET* embryo transfer, *IVF* in vitro fertilization, *ICSI* intracytoplasmic sperm injection, *CET* cryo-thawed embryo transfer, *HRT* hormone replacement therapy

### Metabolic indicators of males grouped by FPG

The metabolic indicators of interest for men are listed in Table 2. The values of metabolic indicators were evaluated by Ks-test. The values of ALT (U/L) [38.0 (26.0–64.0) vs. 28.0 (21.0–40.0), *P* = 0.010], TC (mmol/L) [5.5 ± 1.1 vs. 4.9 ± 0.9, *P* = 0.001], TG (mmol/L) [3.3 ± 1.8 vs. 1.5 (1.1–2.1), *P* < 0.001], were significantly higher in the FH group when compared with the Con group. Whereas, male AST (U/L) [24.0 (19.0–39.0) vs. 21.8 (18.0–27.0), *P* = 0.062], HDL (mmol/L) [1.1 ± 0.2 vs. 1.1 (1.0–1.2), *P* = 0.192], LDL (mmol/L) [3.4 ± 1.2 vs. 3.1 (2.5–3.6), *P* = 0.272], Cr (umol/L) [73.4 ± 10.9 vs. 74.3 ± 11.1, *P* = 0.363], and UA (umol/L) [418.1 ± 114.7 vs. 403.0 (358.0–474.0), *P* = 0.334] were not statistically significant between the two groups.

**Table 2:** Metabolic indicators of male grouped by FPG

Characteristic	FH (*n* = 29)	Con (*n* = 341)	*P-*value
FPG (mmol/L)	6.72 (6.50–7.97)	5.10 (4.84–5.40)	< 0.001*
AST (U/L)	24.0 (19.0–39.0)	21.8 (18.0–27.0)	0.062
ALT (U/L)	38.0 (26.0–64.0)	28.0 (21.0–40.0)	0.010*
TC (mmol/L)	5.5 ± 1.1	4.9 ± 0.9	0.001*
TG (mmol/L)	3.3 ± 1.8	1.5 (1.1–2.1)	< 0.001*
HDL (mmol/L)	1.1 ± 0.2	1.1 (1.0–1.2)	0.192
LDL (mmol/L)	3.4 ± 1.2	3.1 (2.5–3.6)	0.272
Cr (umol/L)	73.4 ± 10.9	74.3 ± 11.1	0.363
UA (umol/L)	418.1 ± 114.7	403.0 (358.0–474.0)	0.334

Data are shown as median (IQR) or n (%)

*P < 0.05 compared with the control group

*FPG* fasting plasma glucose, *AST* aspartate aminotransferase, *ALT* alanine aminotransferase, *TC* total cholesterol, *TG* triglycerides, *HDL* high-density lipoprotein cholesterol, *LDL* low-density lipoprotein cholesterol, *Cr* creatinine, *UA* uric acid. The cut-off points of each metabolic index are recommended by the consensus of Chinese experts

### Effects of male FH on CET clinical outcomes of singleton pregnant women

**Fig. 2:**
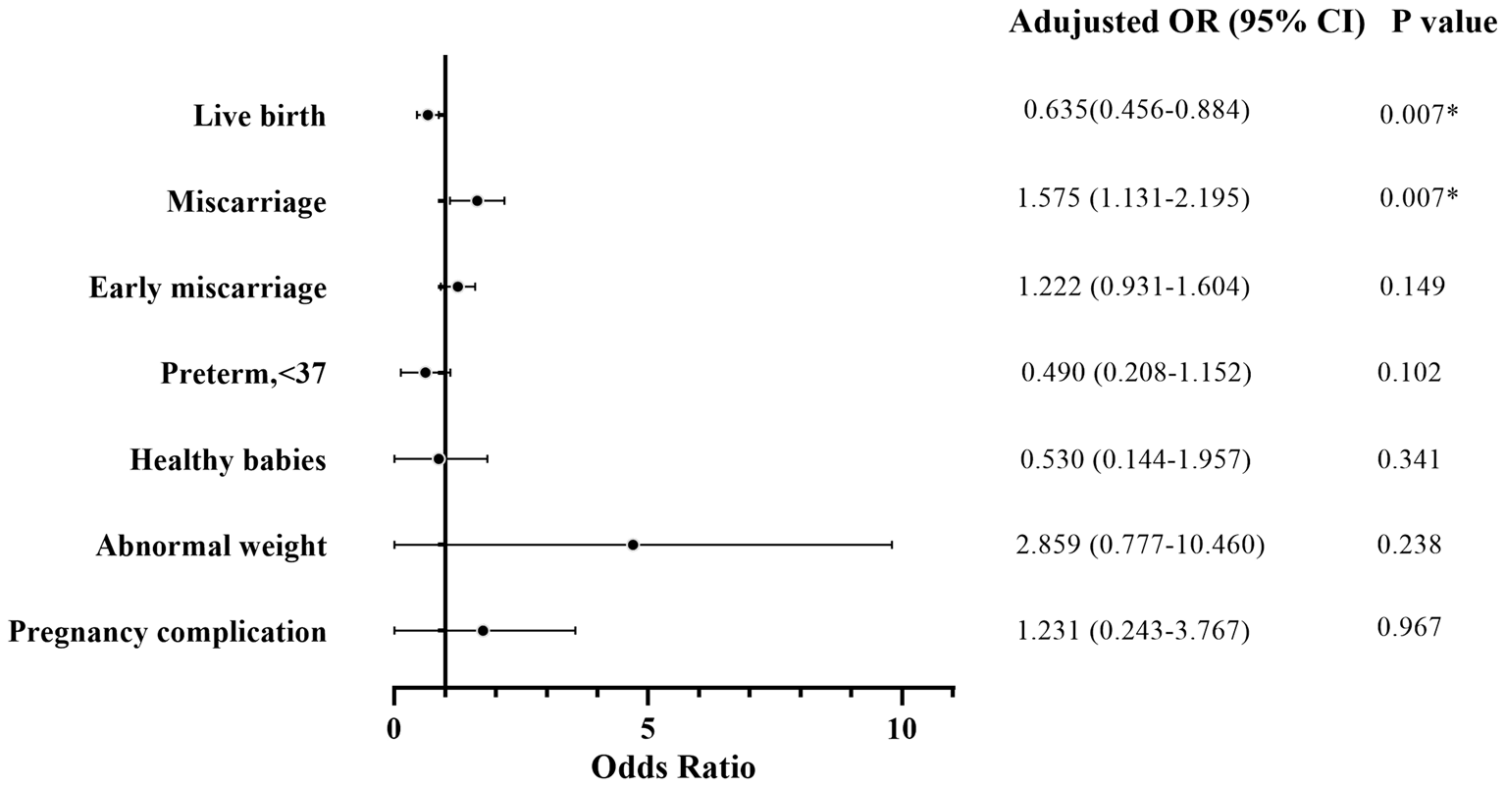
Forest plot of the effect of male FH on pregnancy outcome. It shows the effect of male FH on pregnancy outcomes (*Y*-axis), adjusted for potential confounders, including age, BMI, ALT, HDL and TG level of men, and age and BMI of women. *CI* confidence interval, *P indicated statistical significance

The obstetric and neonatal outcomes of the patients are included in Table 3 and Fig. 2. The LBR was 58.6% (17 of 29) for the FH group and 81.8% (279/341) for the Con group, with an adjusted OR of 0.635 (95% CI 0.456–0.884). The MR was 41.4% (12 of 29) for the FH group and 18.2% (62/341) for the Con group, with an adjusted OR of 1.575 (95% CI 1.131–2.195). Male FH showed an evident impact on the LBR (*P* = 0.007) and MR (*P* = 0.007) after adjusted for age, BMI, HDL, TG, and ALT in men and age in women. The EMR was higher (34.5% vs. 15.8%, *P* = 0.011) in the FH group than the Con group, but this difference disappeared after adjusted for age, BMI, HDL, TG, and ALT in men and age in women [adjusted OR 1.222, 95% CI (0.931–1.604), *P* = 0.149]. The results of the two groups showed no difference in terms of LMR [6.9% vs. 2.3%, adjusted OR 5.215, 95% CI (0.676–35.597), *P* = 0.074].

**Table 3:** Obstetric and neonatal outcomes in the FH versus the control

Outcome	FH (*n* = 29)	Con (*n* = 341)	Unadjusted OR (95% CI)	Adjusted OR (95% CI)	Post-adjustment *P-*value
Live birth^a^ (%)	58.6 (17/29)	81.8 (279/341)	0.315 (0.143–0.693)	0.635 (0.456–0.884)	0.007*
Miscarriage^a^ (%)	41.4 (12/29)	18.2 (62/341)	3.176 (1.444–6.989)	1.575 (1.131–2.195)	0.007*
Early miscarriage	34.5 (10/29)	15.8 (54/341)	2.797 (1.233–6.345)	1.222 (0.931–1.604)	0.149
Late miscarriage	6.9 (2/29)	2.3 (8/341)	3.083 (0.624–15.247)	5.215 (0.676–35.597)	0.074
Gestation weeks at birth (wks)^a^	39 (38.0–40)	39 (38–40)			0.653
Preterm, < 37w (%)	11.8 (2/17)	7.9 (22/279)	1.558 (0.334–7.253)	0.490 (0.208–1.152)	0.102
Healthy babies^a^ (%)	76.5 (13/17)	84.9 (237/279)	0.576 (0.179–1.851)	0.530 (0.144–1.957)	0.341
Birth weight (kg)^a^	3.2 ± 0.7	3.4 (3.1–3.7)			0.325
Abnormal weight	23.5 (4/17)	11.8 (33/279)	2.294 (0.706–7.450)	2.859 (0.777–10.460)	0.238
Very low birth weight, < 1.5 (%)	5.9 (1/17)	1.1 (3/279)			
Low birth weight, < 2.5 (%)	5.9 (1/17)	5.0 (14/279)			
High birth weight, > 4.0 + (%)	11.8 (2/17)	5.7 (16/279)			
Sex of male baby^a^ (%)	58.8 (10/17)	50.9 (142/279)	1.378 (0.510–3.724)	1.548 (0.516–4.640)	0.436
Birth defects (%)	0 (0/17)	0 (0/279)			
Caesarean section^a^ (%)	88.2 (15/17)	58.8 (164/279)	5.259 (1.180–23.442)	5.656 (1.187–25.509)	0.034*
Pregnancy complication^a^ (%)	17.6 (3/17)	14.3 (40/279)	1.280 (0.352–4.656)	1.231 (0.243–3.767)	0.967
Gestational hypertension	11.8 (2/17)	4.7 (13/279)			
Gestational diabetes mellitus	0 (0/17)	2.5 (7/279)			
Placenta previa	0 (0/17)	0.7 (2/279)			
Placental abruption	5.9 (1/17)	0.4 (1/279)			
Premature rupture of membranes	0 (0/17)	6.1 (17/279)			
Postpartum hemorrhage	0	0			

Data are shown as median (IQR) or *n* (%). The outcomes were follow-up data routinely used in reproductive medicine clinics

^a^Adjusted for age, BMI, HDL, TG, ALT in men and age in women. The reference variable within each sub-category has been arbitrarily designated and given the odds ratio of 1

*P indicated statistical significance

In addition, for maternal and infant characteristics, there was no significant difference in the HBR [76.5% vs. 84.9%, *P* = 0.341, adjusted OR 0.530, 95% CI (0.144–1.957)], gestation weeks at birth (wks) [39 (38.0–40) vs. 39 (38–40), *P* = 0.653], PTDR [11.8% vs. 7.9%, *P* = 0.102, adjusted OR 0.490, 95% CI (0.208–1.152)], FTBR [88.2% vs. 92.1%], birth weight (kg) [3.2 ± 0.7 vs. 3.4 (3.1–3.7), *P* = 0.325], birth weight ratio [normal weight rate: 76.5% vs. 88.2%; abnormal weight rate: 23.5% vs. 11.8%, *P* = 0.238, adjusted OR 2.859, 95% CI (0.777–10.460)] or sex of the baby [female:41.2% vs. 49.1%; male: 58.8% vs. 50.9%, *P* = 0.436, adjusted OR 1.548, 95% CI (0.516–4.640)] between the two groups. No birth defects were observed in the present study. The cesarean section rate was much higher in the FH group than in the Con group [88.2% vs. 58.8%, *P* = 0.034, adjusted OR 5.656, 95% CI (1.187–25.509)]. There was no significant difference in the rate of maternal pregnancy complications between the FH and Con group [17.6% vs. 14.3%, *P* = 0.967, adjusted OR 1.231, 95% CI (0.243–3.767)], including gestational hypertension (11.8% vs. 4.7%), gestational diabetes mellitus (0% vs. 2.5%), placenta previa (0% vs. 0.7%), premature rupture of membranes (0% vs. 6.1%) and placental abruption (5.9% vs. 0.4%). No postpartum hemorrhage was observed in our study.

### Multivariate binary logistic regression analysis

A binary logistic regression model was used to analyze the association of male FH with obstetric and neonatal outcomes (Table 3). Univariate logistic regression was used to identify the relevant variables and confounding factors affecting pregnancy outcomes. Variables with univariate *P*-values ≤ 0.10 and potential confounders (Table 4) were included in the multivariate binary logistic regression model. Male FH was an independent predictor for LBR adjusted OR 0.635, 95% CI (0.456–0.884) and in singleton pregnant women with CET after adjustment for age, BMI, HDL, TG, ALT in men, and age in women.

**Table 4:** Multivariate binary logistic regression analysis of the relationship between male FPG and live birth

	Live birth^a^	
	Adjusted OR (95% CI)	*P*-value
FPG (mmol/L)	0.635 (0.456–0.884)	0.007*
HDL (mmol/L)	2.705 (0.896–8.162)	0.078
TG (mmol/L)	0.643 (0.366–1.128)	0.124
ALT (U/L)	1.743 (0.861–3.530)	0.123
Male’s BMI (kg/m^2^)	0.969 (0.888–1.056)	0.473
Male’s age (y)	0.995 (0.927–1.069)	0.896
Female’s age (y)	0.946 (0.865–1.036)	0.232

*FPG* fasting plasma glucose, *ALT* alanine aminotransferase, *TG *triglycerides, *HDL* high-density lipoprotein cholesterol, *BMI* body mass index

^a^Adjusted for age, BMI, HDL, TG, ALT in men and age in women. Multivariate analysis included variables with univariate

*P*-value ≤ 0.1 and confounders those were potential

^*^P indicated statistical significance

## Discussion

In the current retrospective cohort study, the impact of male FH on pregnancy outcomes in couples with ART was investigated. Male FH was found to be an independent risk factor for LBR and MR in women with singleton pregnancies after CET.

Due to changes in lifestyle and dietary habits, MS is on the rise year by year worldwide, and often combined with multiple metabolic diseases that affect the quality of human life. In recent years, an increasing number of studies have begun to focus on the relationship between MS and infertility in men [21–23]. FH as one of the most important metabolic indicators has been noticed to be closely associated with male infertility.

The available researches have shown that male FH is often associated with elevated BMI and metabolic abnormalities, which may contribute to low male fertility and poor embryo quality by impairing steroidogenesis [24, 25]. Most of the study focused BMI or metabolic abnormalities on male reproductive health. A retrospective data analysis showed that sperm count and total concentration were reduced only in overweight or obese men who were metabolically unhealthy, but this study had restricted power and did not report on ART outcomes [26]. What's more, the effect of male hyperglycemia on the outcome of ART has not been documented to date. Only two researchers have attempted to investigate the effect of male hyperinsulinemia on IVF outcomes. One study investigated the effect of hyperinsulinaemia on IVF outcomes, and reported that compared to donors with normal insulin levels, donors pregnancy outcomes in the IVF program [27]. Meanwhile, to investigate the role of insulin in male fertility, the authors did a further study that showed the clinical pregnancy rate in the male normal insulin group was significantly higher than that in the hyperinsulinemia group, suggesting that male hyperinsulinemia had a negative impact on IVF outcomes [4]. Although there were important discoveries revealed by these studies, there were also limitations, first, all these studies had small sample sizes, second, they lacked analysis of confounding factors in ART treatment such as female factors, third, they hadn’t the maternal and neonatal safety assessment, last, male insulin levels were not the essential laboratory indicators to monitor before routine ART treatment. Overall, larger sample sizes including more clinical parameters researches were needed to further validate the effect of male hyperglycemia on ART pregnancy outcomes. FH in men, as a more accessible and visual indicator than insulin, may be more beneficial for timely screening and identification in our routine clinical ART practice. What's more, our primary focus in ART is on clinical live birth outcomes. We strictly selected intrauterine clinical pregnancies after transfer of high-quality embryos as the starting point for our study which helped us to some extent to exclude the effect of embryo and maternal endometrial receptivity on the outcome of live births on ART to better observe the effects of male hyperglycemia on live birth outcomes after clinical pregnancy. Our present study indicated that male FH was an independent risk factor for LBR and MR in singleton pregnancy women after CET. To better control the influence of female factors and other confounding factors, strict inclusion and exclusion criteria were set in present study, we selected women with normal BMI (18.5 ≤ BMI < 25.0) and excluded women with a history of recurrent miscarriages or other autoimmune and metabolic diseases which contributes to excluding some female confounding factors that might be associated with poor pregnancy outcomes. Although there was a significant difference in age between the two groups of women in the univariate analysis of this study, after adjusting for female age as a covariate in logistic regression, we still found that male FH was an independent risk factor for LBR.

In addition, several recent studies on male MS confirmed that obesity and abnormal metabolic indicators in men may impair their reproductive function [21, 26, 28]. Considering the possible influence of other metabolic indicators in men on ART outcomes, we also included these metabolic indicators (ALT, TG, HDL, BMI) that may interfere with the outcomes in the univariate analysis. After correcting for these covariates, it was suggested that even if the male partner did not meet the diagnostic criteria for MS, the mere presence of male FH could affect the LBR and MR.

In our study, we found that the overall MR of the FH group was significantly higher than that of the control group, and the paternal FH remained an independent risk factor for MR after adjusting confounding factors. However, the current findings could not clarify whether male FH had an effect on EMR or LMR owing to the small sample size. Given the large OR value of LMR, we speculate that the effect of FH on LMR may be greater in men after expanding the sample size. What was also interesting in our study was that there was no significant difference in pregnancy complications, except for the higher rate of cesarean delivery in the FH group. This may be partly due to the older age of the women and the fact that the fetus was more precious to them, so more women in the FH group had indications for cesarean delivery or opted for cesarean delivery.

The consensus recommendation of Chinese experts was used as the cut-off criteria for metabolic indicators in men, in which BMI ≥ 25.0 (kg/m^2^) is considered as a risk factor for metabolic syndrome (Table 2) [14, 15]. In our opinion, such criteria are more applicable to the Chinese male population compared with metabolic indicators (BMI, waist circumference, blood pressure, TG, HDL, and FPG) in the NCEP ATP III criteria [29]. Therefore, these findings may not be extrapolated to all ethnic groups. Furthermore, we defined FH when FPG was > 6.1 mmol/L. However, a single isolated episode of FH is not diagnostic of DM [14]. Further investigations are still needed to explore the impact of glucose abnormalities, such as impaired glucose tolerance (IGT) and impaired fasting glucose (IFG), on ART outcomes. In addition, waist circumference and blood pressure are rarely used as routine tests for infertility, but these indicators should actually receive more attention in future investigations.

Although this was a retrospective study, strict inclusion and exclusion criteria were performed to control the confounding factors in the study. We found that male FH did act as an independent risk factor for a lower LBR and higher MR than normoglycemic individuals in CET cycles with singleton intrauterine pregnancies. Despite this result has momentous implications for our efforts to further refine the treatment and outcomes of ART, the effects and molecular mechanisms of male FH on sperm and embryo quality remain unclear. Further research, including basic animal model testing, larger clinical studies and long-term follow-up of offspring, are needed to explore the role of male FH in reproductive health.

## Limitations

Our study has several limitations. Firstly, the statistical power may be weakened by the small sample size. Prospective or randomized controlled studies are needed to further prove our study. Secondly, the study did not describe information on the semen quality and embryos transferred. The effects of male FH on sperm and embryo quality, as well as the related parameters still need further investigations.

## Conclusion

In present study, it was found that male FH is an independent risk factor for lower LBR and higher MR in singleton intrauterine pregnancy women with CET cycles. The biological role of women in fertility and pregnancy outcome is of great interest but the role of men is underestimated. According to present results, it is reasonable to recommend that male patients receive a systematic health assessment and intervention (including diet, lifestyle habits, hyperglycemia, and other disease interventions) before ART treatment.

## Data Availability

Not applicable.

## Abbreviations

ART: Assisted reproductive technology
IVF: In vitro fertilization
ICSI: Intracytoplasmic sperm injection
ET: Embryo transfer
CET: Cryo-thawed embryo transfer
COH: Controlled ovarian hyperstimulation
HRT: Hormone replacement therapy
TESE: Cycles with testicular sperm extraction
TESA: Testicular sperm aspiration
FPG: Fasting plasma glucose
FH: Fasting hyperglycemia
MS: Metabolic syndrome
DM: Diabetes mellitus
ALT: Alanine aminotransferase
AST: Aspartate aminotransferase
TC: Total cholesterol
TG: Triglycerides
HDL: High-density lipoprotein cholesterol
LDL: Low-density lipoprotein cholesterol
Cr: Creatinine
UA: Urea nitrogen
BMI: Body mass index
LBR: Live birth rate
EMR: Early miscarriage rate
LMR: Late miscarriage rate
PTDR: Preterm delivery rate
FTBR: Full-term birth rate
HBR: Healthy baby rate
